# Chronic Type A Aortic Dissection Repair in a Double Lung Transplant Recipient

**DOI:** 10.1055/s-0041-1732400

**Published:** 2021-12-28

**Authors:** Neeraj Kamat, Ragheb Traify, Brian Williams, Ioannis Dimarakis

**Affiliations:** 1 Department of Cardiothoracic Transplantation and Mechanical Circulatory Support, Wythenshawe Hospital, Manchester, United Kingdom; 2 Division of Cardiovascular Sciences, University of Manchester, Manchester, United Kingdom

**Keywords:** lung transplantation, chronic aortic dissection, Stanford Type A aortic dissection

## Abstract

A 69-year-old man presented with a chronic Stanford Type A aortic dissection (CTAAD). The patient had undergone bilateral sequential lung transplantation 15 years prior for α-1-antitrypsin deficiency. We describe the management of CTAAD in the context of lung transplantation from the surgical and anesthetic perspectives.

## Introduction

Surgical intervention for chronic Stanford Type A aortic dissection (CTAAD) has been shown to be associated with increased morbidity and mortality rates. We report successful surgical treatment for CTAAD in a bilateral lung transplant recipient with immunosuppressant-induced nephropathy.

Numerous surgical and anesthetic considerations arise in the setting of previous lung transplantation. Redo surgery with pulmonary allografts potentially crossing the midline in an immunocompromised patient requires meticulous chest entering and mediastinal dissection. Cannulation and venting strategy must take into consideration previous cannulation sites and “neohilums.”

From the anesthetic point of view, apart from perioperative stress-dose steroids and prophylactic antimicrobial administration, treatment must be directed toward prophylactic ventilatory strategies (given the lack of native lymphatic drainage), fluid management, with intraoperative dialysis while on cardiopulmonary bypass (CPB), and near patient coagulation testing coupled with near patient products.

## Case Presentation



**Video 1** Transesophageal echocardiogram images of descending thoracic aorta in short axis depicting further fenestrations between true and false lumens.


**Fig. 1:**
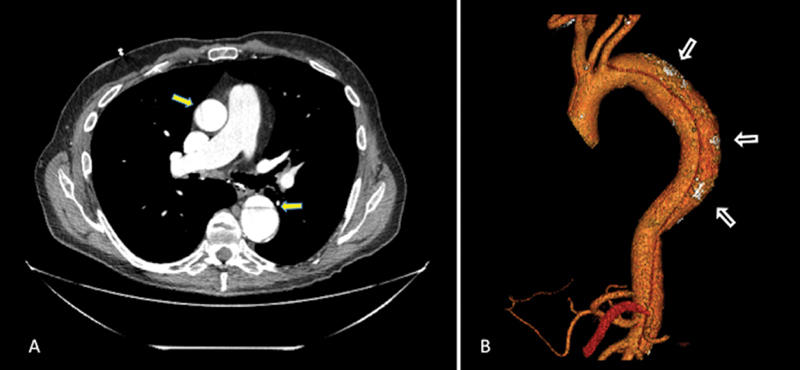
( **A** ) Contrast-enhanced axial computed tomography image demonstrating an intimal flap within the ascending, as well as descending thoracic aorta diagnostic of a Stanford Type A dissection (block arrows). ( **B** ) Computed tomography, three-dimensional reconstruction image. The extent of the dissection can be seen spanning the entire length of the descending thoracic and abdominal aorta, with visceral arterial supply originating from the true lumen. Outer wall calcification of the false lumen can also be seen (open arrows).

A 69-year-old male patient presented to his local hospital with insidious onset of chest pain for the past 10 days. He was hemodynamically stable without features of cardiogenic shock. As initial investigations detected elevated D-dimer levels, computed tomographic pulmonary angiography was performed to rule out pulmonary embolism that was excluded, but incidental findings of a Type A aortic dissection were seen. Subsequent computed tomography aortogram depicted a Type A aortic dissection originating in the ascending aorta, involving the innominate artery, and extending to the aortic bifurcation. The proximal ascending aorta and aortic root were not involved. All visceral arteries were seen to arise from the true lumen. Despite the subacute presentation, signs of chronicity were seen on imaging, including a thick and straight dissection flap alongside a dilated false lumen with outer wall calcification ( Fig. 1 ). On echocardiography, biventricular size and function were preserved with no valvular abnormality documented.

The patient had undergone bilateral sequential lung transplantation 15 years prior for α-1-antitrypsin deficiency. A clamshell incision had been used for access with CPB via central cannulation. The patient had regularly attended the lung transplant outpatient clinic since his original discharge, having developed a degree of immunosuppressant-induced nephropathy and hypertension.

The patient underwent urgent operative repair through median sternotomy (oscillating saw) with attention to minimize trauma to the pulmonary allografts during dissection. Due to involvement of the innominate artery, the left axillary artery was cannulated via a 8-mm synthetic graft, and venous return was established with a percutaneous right femoral venous cannula. Pulmonary artery and left apical venting were used to avoid dissection near the right hilum. Under moderate hypothermia, circulatory arrest was instituted with bilateral selective antegrade cerebral perfusion.

**Fig. 2:**
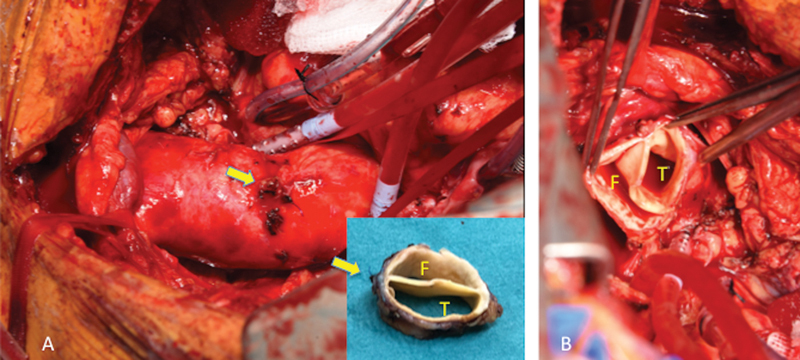
Intraoperative images. ( **A** ) The ascending aorta dissected out; the intimal tear was located in proximity to the site of the previous aortic cannulation (block arrows) ( **B** ) The chronic dissection flap seen separating the true (T) and false (F) lumens at the level of the aortic arch.

Interestingly, the primary intimal tear was located in proximity to the site of the previous aortic cannulation, with only distal progression of the flap and no involvement of the aortic root ( Fig. 2 ). Multiple fenestrations were seen on transesophageal echocardiography in the descending thoracic aorta ( Video 1 , available in the online version). Open distal anastomosis with a Gelweave Ante-Flo graft (Vascutek Ltd., Renfrewshire, Scotland, United Kingdom) was performed. Full flow was restored through the side arm of the graft. The proximal anastomosis was performed above the sinotubular junction.

The ventilatory strategy employed optimized positive end expiratory pressure (PEEP) and tidal volumes of 6 to 8 mL/kg, ensuring peak pressures lower than 25 cm H _2_ 0. A combination of low tidal volume and PEEP was used during CPB to minimize atelectasis and reduce the inflation pressures required for reinflation prior to separation from CPB. Our postoperative strategy was to aim for early extubation to minimize total ventilation time.

Close attention was given to fluid management given the risk of excess fluid to the lungs in the context of renal impairment. The CPB circuit was primed with human albumin solution to improve colloid oncotic pressure and minimize third space losses. We elected to use filtration on CPB to prevent inadvertent fluid overload and limit metabolic derangement.

Postoperatively, extubation was performed on the first postoperative day and following an uncomplicated course the patient was discharged on the 12th postoperative day after reestablishing maintenance immunosuppressive treatment.

Histological examination of the resected segments of aortic wall showed extensive dissection, moderate myxoid change, elastic fiber disruption, patchy fibrosis, and mild inflammation in the adventitia.

## Discussion

Limited information is available regarding the natural history of chronic Type A aortic dissections, with recent data indicating that risk of aortic rupture or sudden death correlates with age and baseline aortic diameter. 1 Furthermore, a paucity of literature exists on outcomes of conventional cardiac surgery following lung transplantation. 2

Hermsen et al 3 describe successful repair of an acute Type A aortic dissection in a lung transplant recipient. Similar to our case, the patient did display immunosuppressant-induced end-stage renal disease and hypertension. One further case of iatrogenic cannulation-related Type A aortic dissection at the time of transplantation has been reported to our knowledge. 4 Due to the atypical clinical presentation of our patient, radiological assessment was particularly useful as we did anticipate an element of chronicity. 5

We opted for antegrade perfusion via the axillary artery as that is our first-line approach in complex aortic surgery to minimize the risks of distal embolization, limb ischemia, and retrograde dissection. Our preferred cannulation site is the right axillary artery pending review of preoperative imaging. Awareness of “neohilum” anatomy is also pertinent to avoid inadvertent injuries to the pulmonary allografts. It must be said that the senior authors have aortic, as well as cardiothoracic transplant practices that aided decision-making.

Chronic expansion of the false lumen with exposure of subendothelial collagen tissue and stagnation of a high volume of blood in the false lumen are contributing factors to the rarely seen disseminated intravascular coagulation of aortic dissection. 6 Massive transfusion of clotting products would have potentially posed a significant additional risk to the transplanted lungs, through both fluid balance and potential for transfusion-related acute lung injury. Accordingly, we made use of near patient coagulation testing coupled with near patient products, such as prothrombin complex concentrate (PCC) and fibrinogen complex concentrate, to allow a rapid response to coagulopathy. This was combined with full-dose Hammersmith protocol aprotinin and timely transfusion of platelets following administration of protamine. One of the limitations of PCC is that most formulations lack von Willebrand factor (vWf). This was accounted for by using desmopressin which releases vWf from the endothelium and improves platelet function and clotting.

Local α-1-antitrypsin deficiency in the human ascending aorta has been suggested to lead to proteolytic damage easing aortic dissection. 7 We suspect that primary pathology in combination with long-term immunosuppression in certain transplant recipients may precipitate aortic pathology via a variety of mechanisms, including potential harmful effects on collagen formation and connective tissue strength. This corroborates with several published reports of aortic dissection following other types of solid organ transplantation. 8

Meticulous preoperative planning remains the mainstay of approaching such cases. Added consideration should be given to the anatomical and physiological specifics of lung transplantation from the surgical, as well as anesthetic perspective.

## References

[1] Kim W K Park S J Kim H J Kim H J Choo S J Kim J B The fate of unrepaired chronic type A aortic dissection J Thorac Cardiovasc Surg 2019 158 04 996 1.004E6, e3 30578057 10.1016/j.jtcvs.2018.11.021

[2] Banack T Ziganshin B A Barash P Elefteriades J A Aortic valve replacement for critical aortic stenosis after bilateral lung transplantation Ann Thorac Surg 2013 96 04 1475 1478 24088467 10.1016/j.athoracsur.2013.01.022

[3] Hermsen J L Madathil R Berfield K S Li K Smith J W Mulligan M S Successful repair of acute Type A aortic dissection 15 years following bilateral lung transplantation J Card Surg 2016 31 01 72 73 26585692 10.1111/jocs.12671

[4] Fleck T Ehrlich M Czerny M Wolner E Grabenwoger M Grimm M Intraoperative iatrogenic type A aortic dissection and perioperative outcome Interact Cardiovasc Thorac Surg 2006 5 01 11 14 17670501 10.1510/icvts.2005.114900

[5] Orabi N A Quint L E Watcharotone K Nan B Williams D M Kim K M Distinguishing acute from chronic aortic dissections using CT imaging features Int J Cardiovasc Imaging 2018 34 11 1831 1840 29915877 10.1007/s10554-018-1398-x

[6] Gatate Y Masaki N Sato A Tranexamic acid controlled chronic disseminated intravascular coagulation associated with aortic dissection and patent false lumen for three years Intern Med 2017 56 08 925 929 28420841 10.2169/internalmedicine.56.7499 PMC5465409

[7] Schachner T Golderer G Sarg B The amounts of alpha 1 antitrypsin protein are reduced in the vascular wall of the acutely dissected human ascending aorta Eur J Cardiothorac Surg 2010 37 03 684 690 19709897 10.1016/j.ejcts.2009.07.025

[8] Cron D C Coleman D M Sheetz K H Englesbe M J Waits S A Aneurysms in abdominal organ transplant recipients J Vasc Surg 2014 59 03 594 598 24246534 10.1016/j.jvs.2013.09.049

